# 基于暴露组-脂质组关联研究的代谢相关脂肪性肝病血清中外源性化学物质的风险分析

**DOI:** 10.3724/SP.J.1123.2023.12014

**Published:** 2024-02-08

**Authors:** Qianqian CHEN, Lei YOU, Pengwei GUAN, Chengnan FANG, Wangshu QIN, Xinyu LIU, Guowang XU

**Affiliations:** 1.中国科学院大连化学物理研究所, 中国科学院分离分析化学重点实验室, 辽宁省代谢组学重点实验室, 辽宁 大连 116023; 1. Liaoning Province Key Laboratory of Metabolomics, CAS Key Laboratory of Separation Science for Analytical Chemistry, Dalian Institute of Chemical Physics, Chinese Academy of Sciences, Dalian 116023, China; 2.中国科学院大学, 北京 100049; 2. University of Chinese Academy of Sciences, Beijing 100049, China; 3.中国医科大学, 辽宁 沈阳 110122; 3. China Medical University, Shenyang 110122, China

**Keywords:** 代谢相关脂肪性肝病, 暴露组学, 脂质组学, 组学关联研究, metabolic associated fatty liver disease (MAFLD), exposomics, lipidomics, omics-wide association study

## Abstract

代谢相关脂肪性肝病是当前常见的一种肝脏疾病,在世界范围内的患病率高达25%,严重危害人类健康并对社会造成巨大的经济负担。越来越多的研究表明慢性非传染性疾病的发生是环境暴露与遗传因素共同作用的结果,环境污染是其不可小觑的健康风险因素。为了探究环境暴露对代谢相关脂肪性肝病风险及暴露效应,本研究利用超高效液相色谱-串联质谱(UHPLC-MS/MS)的靶向暴露组学和超高效液相色谱-高分辨质谱(UHPLC-HRMS)的非靶向脂质组学技术分别分析了代谢相关脂肪性肝病患者血清中外源性化学物质的暴露特征和内源性脂质代谢物的扰动,结合暴露组-脂质组关联分析,在脂代谢水平上探究环境暴露引起的代谢相关脂肪性肝病的风险及暴露效应。研究发现,代谢相关脂肪性肝病患者体内外源性化学物质与代谢相关脂肪性肝病的风险增加有关,其中氟虫腈砜(fipronil sulphone)、马拉硫磷二羧酸(malathion dicarboxylic acid)和邻苯二甲酸单环己酯(monocyclohexyl phthalate)与单纯性脂肪肝风险呈正相关,氟虫腈砜(fipronil sulphone)、安赛蜜(acesulfame potassium)、全氟辛酸(PFOA)、全氟壬酸(PFNA)、全氟十一酸(PFUnDA)和4-羟基二苯甲酮(4-hydroxybenzophenone)以及3,5-二叔丁基-4-羟基苯甲酸(DBPOB)与合并代谢性疾病的脂肪肝风险呈正相关。单纯性脂肪肝患者和合并代谢性疾病的脂肪肝患者的脂代谢发生了显著的改变,神经酰胺(Cer)、甘油三酯(TG)和甘油二酯(DG)显著升高,这些DG和TG的酰基碳数分别为32~40和35~60,且两者均表现为多不饱和的脂质分子的变化为主。大多数的脂质效应标志物与外源性化学物质残留呈正相关,并与疾病风险增加有关。本研究可以为环境化学物质暴露与代谢相关脂肪性肝病的关联与机制研究提供科学依据。

## Abstract (en)

Metabolic associated fatty liver disease (MAFLD) is a common liver disease with a prevalence of up to 25%; it not only adversely affects human health but also aggravates the economic burden of society. An increasing number of studies have suggested that the occurrence of chronic noncommunicable diseases is affected by both environmental exposures and genetic factors. Research has also shown that environmental pollution may increase the risk of MAFLD and promote its occurrence and development. However, the relationship between these concepts, as well as the underlying exposure effects and mechanism, remains incompletely understood. Lipidomics, a branch of metabolomics that studies lipid disorders, can help researchers investigate abnormal lipid metabolites in various disease states. Lipidome-exposome wide association studies are a promising paradigm for investigating the health effects of cumulative environmental exposures on biological responses, and could provide new ideas for determining the associations between metabolic and lipid changes and disease risk caused by chemical-pollutant exposure. Hence, in this study, targeted exposomics and nontargeted lipidomics studies based on ultra-high performance liquid chromatography-tandem mass spectrometry (UHPLC-MS/MS) and ultra-high performance liquid chromatography-high resolution mass spectrometry (UHPLC-HRMS) were used to characterize exogenous chemical pollutants and endogenous lipid metabolites in the sera of patients with MAFLD and healthy subjects. The results demonstrated that fipronil sulfone, malathion dicarboxylic acid, and monocyclohexyl phthalate may be positively associated with the disease risk of patients diagnosed as simple fatty liver disease (hereafter referred to as MAFLD(0)). Moreover, fipronil sulfone, acesulfame potassium, perfluorooctanoic acid (PFOA), perfluorononanoic acid (PFNA), perfluoroundecanoic acid (PFUnDA), 4-hydroxybenzophenone, and 3,5-di-tert-butyl-4-hydroxybenzoic acid (DBPOB) may be positively associated with the disease risk of patients diagnosed as fatty liver complicated by single or multiple metabolic disorders. Association analysis was carried out to explore the lipid metabolites induced by chemical residues. Triglyceride (TG) and diglyceride (DG) were significantly increased in MAFLD and MAFLD(0). The numbers of carbons of significantly changed DGs and TGs were mainly in the ranges of 32-40 and 35-60, respectively, and both were mainly characterized by changes in polyunsaturated lipids. Most of the lipid-effect markers were positively correlated with chemical residues and associated with increased disease risk. Our research provides a scientific basis for studies on the association and mechanism between serum chemical-pollutant residues and disease outcomes.

脂肪肝是一种由多种原因导致肝细胞内脂肪堆积过多的疾病,一般分为酒精性脂肪肝和非酒精性脂肪肝两大类。非酒精性脂肪肝是一种无过量饮酒和其他明确的肝脏损伤因素导致的,以肝脏实质细胞脂肪变性为特征的疾病^[1]^。2020年初,国际专家小组发布共识声明^[2]^,将非酒精性脂肪性肝病(nonalcoholic associated fatty liver, NAFLD)改名为代谢相关脂肪性肝病(metabolic associated fatty liver disease, MAFLD),该病全球患病率日益攀升,已高达25%^[3]^。

流行病学证据表明,MAFLD是一种多系统疾病^[4]^,不仅会造成终末期肝病^[5]^、原发性肝癌^[5]^,还与2型糖尿病^[6]^、心血管疾病^[7]^、肥胖^[8]^及高尿酸血症^[9]^等密切相关。近年来,越来越多的研究显示慢性代谢性疾病不单受到遗传因素的控制,还与周围环境的暴露密不可分,包括MAFLD的多种疾病均与环境暴露和遗传等多种因素联合作用相关^[10]^。有研究发现,环境污染物(如全氟辛烷磺酸)暴露与MAFLD患者肝脏脂质谱的改变有关,外源性化学物质可能是MAFLD发生和发展的重要影响因素^[11]^。

肝脏是体内新陈代谢的中心器官,当脂质在肝脏内的生成与消耗平衡被扰乱时,就会促进MAFLD的形成,因此MAFLD与肝脏内的脂质代谢紧密关联^[12]^。脂质组学旨在大规模定性和定量研究脂类化合物,能准确全面地提供生物样品在不同生理条件下的全脂信息谱图,在解释脂质代谢相关疾病的发病机制方面发挥了重要作用^[13]^。现有脂肪肝代谢组学和脂质组学研究的证据表明,氨基酸、花生四烯酸、胆汁酸以及脂质代谢途径在MAFLD患者中显著变化,尤其是脂质代谢^[14]^。此外,研究还发现,与对照组相比,NAFLD患者血浆中多种甘油二酯(DG)特异性增加^[15]^。与代谢组类似,脂质组能有效反映内外界因素引起的脂代谢紊乱,有助于从脂代谢视角深入了解环境化学物质暴露与MAFLD疾病风险关联与作用机制。

因此,本研究拟采用基于超高效液相色谱-三重四极杆质谱(UHPLC-MS/MS)的靶向暴露组学定量血清中外源性化学物质残留,采用超高效液相色谱-高分辨质谱(UHPLC-HRMS)的非靶向脂质组学技术获取MAFLD患者血清脂质谱,通过暴露组关联研究确定外源性化学物质残留与代谢相关脂肪性肝病患者的风险关系,通过脂质组关联研究结合中间相遇原则揭示外源性化学物质残留引起与疾病风险相关的脂代谢扰动,为全面深入了解代谢相关脂肪性肝病的发病机制提供理论基础。

## 1 实验部分

### 1.1 仪器、试剂与材料

台式高速冷冻离心机(日本HITACHI公司);振荡器(美国Avantor公司);真空离心浓缩仪(美国LABCONCO公司); 96孔氮吹仪(中国奥盛仪器有限公司);氮气发生器(中国森奥电器有限公司);超纯水系统(美国Millipore公司)。

甲醇、乙腈和异丙醇(色谱纯)购于德国Merck公司;甲酸购于中国J&K Scientific公司;甲基叔丁基醚(MTBE)和乙酸铵(粉末,纯度99%)购于美国Sigma-Aldrich公司。脂质组学内标购自美国Avanti Polar Lipids公司。254种化学物质标准品(包含酚类、全氟化合物、紫外线吸收剂、除草剂、杀虫剂、杀菌剂、兽药、食品添加剂等)和暴露组学内标购于Sigma-Aldrich、J&K Scientific等公司。磷脂去除96孔板Phree^TM^Phospholipid Removal Plates购于美国Phenomenex公司。

### 1.2 样本采集

本研究涉及的337例样本采自锦州医科大学第一附属医院,以诊断为MAFLD的患者为受试对象,采集血清样本并收集受试者的年龄、性别、体重、血糖、血脂及肾功能等生化指标信息。

本研究得到锦州医科大学附属第一医院医学研究伦理委员会的批准(KYLL202084),并按照1975年修订的1964年赫尔辛基宣言的指导方针进行。所有受试者均签署知情同意书。

### 1.3 非靶向脂质组学分析^[16]^

样本预处理 采用研究组建立的甲醇-MTBE-水溶剂体系提取血清中的脂质。简述如下:量取20 μL血清样本,加入240 μL含内标(质量浓度见表1)的甲醇溶液。涡旋后,加入800 μL MTBE,室温振荡后加入水分层,涡旋离心后取上清液冻干。用二氯甲烷-甲醇(2∶1, v/v)复溶,含5 mmol/L乙酸铵的乙腈-异丙醇-水(65∶30∶5, v/v/v)的混合液稀释后进样分析。质量控制(QC)样本由相同体积的所有待检测样本混合而成,用来监测和评价样本分析过程中数据的稳定性,其预处理操作同实际样本。

**表1:** 内标化合物的信息

IS	ESI mode	*C*(IS)/ (μg/mL)	Platform
FFA16∶0-d_3_	ESI^-^	0.67	lipidomics
FFA18∶0-d_3_	ESI^-^	0.67	lipidomics
LPC 19∶0	ESI^+^	0.33	lipidomics
PC 38∶0	ESI^-^	0.67	lipidomics
PE 30∶0	ESI^-^	0.33	lipidomics
SM(d18∶1/12∶0)	ESI^+^	0.17	lipidomics
Cer(d18∶1/17∶0)	ESI^-^	0.17	lipidomics
Coumaphos-d_10_	ESI^+^	0.5	exposomics
Dimethoate-d_6_	ESI^+^	2.5	exposomics
Imidacloprid-d_4_	ESI^+^	5	exposomics
Prometryn-d_4_	ESI^+^	1	exposomics
Linuron-d_6_	ESI^+^	5	exposomics
Metalaxyl-d_6_	ESI^+^	0.5	exposomics
Myclobutanil-d_9_	ESI^+^	1	exposomics
Carbendazim-d_4_	ESI^+^	0.5	exposomics
Sulfamerazine-^13^C_6_	ESI^+^	2.5	exposomics
Sulfamethazine-(phenyl-^13^C_6_)	ESI^+^	1.5	exposomics
Metronidazole-^13^C_2_,^15^N_2_	ESI^+^	5	exposomics
Clenbuterol-d_9_	ESI^+^	5	exposomics
Furaltadone-d_5_	ESI^+^	10	exposomics
2,4-D-^13^C_6_	ESI^-^	5	exposomics
Triclosan-^13^C_12_	ESI^-^	15	exposomics
Fipronil-d_4_	ESI^-^	0.5	exposomics
Chloramphenicol-d_5_	ESI^-^	2	exposomics
PFOA-^13^C_8_	ESI^-^	3	exposomics
PFOS-^13^C_8_	ESI^-^	3	exposomics

FFA16∶0-d_3_: palmitic acid-16,16,16-d_3_; FFA18∶0-d_3_: stearic acid-18,18,18-d_3_; LPC: lysophosphatidylcholine; PC: phosphatidylcholine; PE: phosphatidylethanolamine; SM: sphingomyelin; Cer: ceramide; PFOA: perfluorooctanoic acid; PFOS: perfluorooctanesulfonate.

色谱条件 脂质组学分析采用Vanquish超高效液相色谱-Q Exactive^TM^质谱系统(美国Thermo Fisher Scientific公司)。采用ACQUITY UPLC BEH C8色谱柱(100 mm×2.1 mm, 1.7 μm,美国Waters公司)在柱温60 ℃进行色谱分离。以含10 mmol/L乙酸铵的乙腈-水溶液(60∶40, v/v)和含10 mmol/L乙酸铵的异丙醇-乙腈溶液(90∶10, v/v)为流动相进行梯度洗脱。

质谱条件 ESI源正离子检测模式下,MS全扫描范围为*m/z* 300~1100,喷雾电压3.50 kV;负离子检测模式下,MS全扫描范围为*m/z* 160~1600,喷雾电压为3.00 kV。毛细管温度300 ℃,辅助加热器温度350 ℃,鞘气和辅助气流速分别为45和10 arb。全扫描MS和数据依赖型二级质谱(dd-MS^2^)两种模式下的分辨率分别设置为120000和30000。以QC样本进行dd-MS^2^扫描用于脂质定性。

### 1.4 靶向暴露组学定量分析^[17]^

样本预处理 利用研究组建立的基于96孔除磷脂板的暴露组学前处理方法。简述如下:量取50 μL血清样本,加入200 μL混合内标溶液(内标浓度见表1,内标溶于乙腈)及50 μL乙腈。振荡混合后,利用接收板收集提取液,并利用氮气吹干。以50 μL甲醇-水(1∶1, v/v)复溶,振荡混合并经过0.22 μm过滤后进样分析。

配制基质混合标准溶液和溶剂混合标准溶液供定量使用,质量浓度梯度如下:200、100、50、25、10、5、2.5、1、0.5、0.25、0.1、0.05、0.025、0.01、0.005、0.0025、0.001 ng/mL。

色谱条件 暴露组学分析采用ExionLC AD超高效液相色谱-Triple Quad^TM^6500^+^质谱系统(美国AB SCIEX公司)。采用ACQUITY UPLC BEH C18色谱柱(50 mm×2.1 mm, 1.7 μm,美国Waters公司),柱温60 ℃,流速0.4 mL/min。以含5 mmol/L乙酸铵的水溶液和含10 mmol/L乙酸铵的甲醇溶液为流动相进行梯度洗脱。

质谱条件 利用ESI源,正、负离子切换检测模式同时扫描。正、负离子检测模式下,喷雾电压分别为5.50 kV和-4.50 kV。温度设定为500 ℃,离子源气1和离子源气2都设为344.74 kPa。分析过程中,插入空白样本(乙腈)和QC样本分别监测分析过程中可能产生的残留及仪器的分析稳定性。每运行完96针,插入2针空白样本冲洗系统(冲洗2 h)。

### 1.5 数据处理与统计分析

利用LipidSearch^TM^4.1软件(美国Thermo Scientific公司)对QC样本的dd-MS^2^数据进行定性,获得精确质荷比、保留时间、脂质类型、脂酰基离子碎片及质量精度等信息,然后通过Xcalibur软件(美国Thermo Scientific公司)对脂质的结构信息以及保留时间进行核对,对未产生特征碎片的脂质根据实验室自建数据库进行靶向提取。

利用SCIEX OS软件(美国AB SCIEX公司)对QC样本中各个化学物质进行积分,根据校正质控样本中获得最小相对标准偏差(RSD)为原则选择合适的内标,并用于实际样本和基质/溶剂加标线性样本的校正。建立加权(权重为1/浓度)线性回归模型拟合校准曲线,定量实际样本中的暴露物。默认使用基质加标线性定量,在基质干扰严重时选用溶剂加标线性定量。在统计分析之前,将定量结果中低于LOQ的检测值替换为LOQ/2值。

利用Mann-Whitney非参数检验对差异代谢物做层次聚类分析,利用调整混杂因素后的Spearman相关性分析和二元逻辑回归分析,考察外源性暴露物、内源性脂质代谢物和代谢相关脂肪性肝病的关系等。

## 2 结果与讨论

### 2.1 临床指标的比较

根据受试者生化指标等信息将疾病组细分为4种情况:(1)只患脂肪肝(以MAFLD(0)表示),共52例;(2)患脂肪肝且合并高尿酸血症、高脂血症、肥胖、糖尿病和高血压任意1种代谢性疾病(MAFLD&1),共121例;(3)患脂肪肝且合并2种代谢性疾病(MAFLD&2),共92例;(4)患脂肪肝且合并3种代谢性疾病(代谢综合征)(MAFLD&3),共35例;(5)患有脂肪肝且合并4种代谢性疾病(MAFLD&4),共7例。此外,研究中纳入30例健康对照(HC)。

代谢性疾病的诊断标准如下:高尿酸血症,尿酸水平男性≥420 μmol/L,女性≥360 μmol/L;高脂血症,满足以下任一条件,甘油三酯水平≥2.3 μmol/L、总胆固醇水平≥6.2 μmol/L、低密度脂蛋白胆固醇水平≥3.4 μmol/L;肥胖,身体质量指数(BMI)≥28;高血压,满足以下任一条件,收缩压≥140 mmHg、舒张压≥90 mmHg;糖尿病,空腹血糖浓度水平≥7.0 μmol/L。与健康对照组相比,代谢相关脂肪性肝病组在性别、体重指数、甘油三酯、高密度脂蛋白胆固醇、体重、收缩压与舒张压等指标存在显著差异,详见表2。

**表2:** 本工作研究对象的详细临床信息

Characteristic	Total		Control		MAFLD(0)		MAFLD&1		MAFLD&2		MAFLD&3		MAFLD&4	
Gender (male/female)	146	/191	14	/16	15	/37	50	/71	45	/47	18	/17	4	/3
Age/years	45.9	±10.6	41.8	±9.0	46.2	±10.0	44.6	±9.4	47.7	±11.4^*^	48.5	±12.7^*^	48.0	±12.8
BMI/(kg/m^2^)	26.6	±3.0	24.0	±1.7	25.5	±1.5^***^	25.9	±2.8^***^	28.0	±3.0^***^	28.4	±2.7^***^	30.8	±4.4^***^
SBP/mmHg	133.5	±18.8	120.0	±11.0	121.0	±12.6	132.4	±17.2^***^	141.8	±21.3^***^	141.7	±12.8^***^	150.3	±10.7^***^
DBP/mmHg	81.9	±11.7	74.6	±8.0	73.6	±8.7	81.9	±11.3^***^	86.4	±11.7^***^	86.8	±10.6^***^	90.1	±8.6^**^
Height/cm	165.5	±8.3	165.9	±7.0	163.2	±7.0	165.3	±8.7	167	±8.6	165.9	±8.6	163.8	±10.4
Weight/kg	73.1	±11.8	65.8	±5.0	67.9	±7.2	71.1	±11.2^*^	78.3	±12.9^***^	78.5	±12.3^***^	82.0	±8.3^**^
GLU/(mmol/L)	5.7	±1.6	5.0	±0.3	5.1	±0.5	5.5	±1.2^***^	6.2	±2.0^***^	6.4	±2.1^***^	8.0	±3.7^**^
TG/(mmol/L)	1.5	±1.0	1.0	±0.3	1.2	±0.4	1.6	±1.0^***^	1.6	±0.9^***^	1.9	±1.0^***^	3.1	±1.9
TC/(mmol/L)	4.8	±0.9	4.3	±0.7	4.3	±0.5	4.9	±0.8^**^	5.0	±0.9^***^	5.3	±0.9^***^	5.4	±0.8
HDL-c/(mmol/L)	1.2	±0.2	1.4	±0.2	1.3	±0.2	1.2	±0.3^***^	1.2	±0.2^***^	1.2	±0.3^***^	1.2	±0.1
LDL-c/(mmol/L)	3.0	±0.7	2.6	±0.6	2.6	±0.5	3.1	±0.7^***^	3.2	±0.8^***^	3.5	±0.7^***^	3.2	±0.7
Hb/(g/L)	144.5	±16.5	143.3	±15.5	139.6	±15.5	144.7	±16.5	145.6	±18.2	147.9	±14.1	150.3	±13.7
Cre/(μmol/L)	66.2	±13.6	65.1	±13.7	62.7	±12.8	66.5	±13.3	66.7	±12.3	67.5	±13.3	79.6	±30.1
UA/(μmol/L)	331.3	±77.1	296.3	±62.6	303.8	±49.6	329.1	±69.4^*^	339.0	±84.2^**^	371.2	±96.1^***^	421.3	±69.2^**^
Urea/(mmol/L)	4.6	±1.1	4.4	±1.0	4.4	±1.0	4.7	±1.1	4.6	±1.1	4.7	±0.9	6.3	±1.5^**^
AST/(U/L)	19.8	±7.9	17.7	±3.8	17.1	±4.8	20.2	±7.6	19.8	±7.5	22.8	±11.2	25.4	±18.7
ALT/(U/L)	25.7	±17.4	17.8	±8.2	19.5	±9.7	27.5	±18.2^**^	27.0	±18.8^*^	30.0	±18.7^**^	37.1	±28.5
AST/ALT	0.9	±0.4	1.1	±0.4	1.0	±0.3	0.9	±0.3^***^	0.9	±0.3^***^	0.9	±0.6^**^	0.7	±0.1

MAFLD(0), MAFLD&1, MAFLD&2, MAFLD&3, MAFLD&4: metabolic associated fatty liver disease with no, one, two, three, four metabolic disorder, respectively; BMI: body mass index; SBP: systolic blood pressure; DBP: diastolic blood pressure; GLU: glucose; TG: triglyceride; TC: total cholesterol; HDL-c: high density lipoprotein cholesterol; LDL-c: low density lipoprotein cholesterol; Hb: hemoglobin; Cre: creatinine; UA: uric acid; AST: aspartate aminotransferase; ALT: alanine aminotransferase. * *p*<0.05, ** *p*<0.01, *** *p*<0.001 compared with health control (HC). *p* values were calculated by nonparametric tests. Data represent mean±SD.

### 2.2 血清中化学物质残留的检测及数据质量评价

暴露物的峰面积经内标校正后,计算质控样本中暴露物含量的RSD,评价仪器的稳定性和数据的重复性。结果显示,96%暴露物含量的RSD<30%,表明数据总体质量良好。QC中RSD<30%的物质线性范围跨越3~4个数量级,具有良好的线性且相关系数平方(*r*^2^)在0.99以上。

本研究检测的254种物质中,167种物质的检出率<30%, 55种物质的检出率>30%, 32种物质未检出。全氟辛酸(PFOA)、全氟己烷磺酸(PFHxS)、全氟庚基磺酸(PFHpS)的检出率为100%。全氟壬酸(PFNA)、氟虫腈砜、全氟十一酸(PFUnDA)、可的松、糠酸莫美他松、皮质醇检出率为99%。此外,全氟戊酸(PFPeA)、噻虫嗪、全氟丁基磺酸(PFBS)等的检出率均在90%以上(见表3)。有研究报道全氟类化合物暴露很普遍^[18]^,在人体中更容易积累。检测频率>30%的物质被纳入后续分析。

**表3:** 检出率大于30%的外源性化学物质

Chemical contaminant	Chinese name	Frequency/%	*C*/(ng/mL)	
Imipramine hydrochloride	盐酸丙咪嗪	46.29	0.101	±0.067
Tritolyl phosphate	磷酸三甲苯酯	83.09	0.128	±0.667
Ethylhexyl dimethyl PABA	二甲基PABA乙基己酯	38.58	0.290	±0.229
Cortisone	可的松	99.41	17.512	±4.279
Cortisol	皮质醇	99.11	36.361	±18.992
Thiamethoxam	噻虫嗪	98.52	15.761	±10.035
5-Formylsalicylic acid	5-甲酰水杨酸	45.99	0.157	±0.110
Lufenuron	虱螨脲	75.37	0.241	±0.147
Dibutyl phosphate	磷酸二丁酯	70.33	0.470	±0.272
Diphenyl phosphate	磷酸二苯酯	64.99	0.473	±0.527
Carbamazepine	卡马西平	37.69	4.786	±64.549
Ampicillin	氨苄青霉素	35.91	12.742	±2.702
Metribuzin	嗪草酮	40.95	1.474	±0.644
Ofloxacin	氧氟沙星	61.42	2.594	±1.632
Bentazone	灭草松	31.45	0.020	±0.024
Perfluoroheptanesulfonic acid	全氟庚基磺酸	100	0.330	±0.248
Bisphenol A	双酚A	71.22	8.346	±14.892
Pentachlorophenol	五氯苯酚	55.49	1.890	±4.426
Methyl 4-hydroxybenzoate	尼泊金甲酯	53.12	7.128	±10.557
Salicylic acid	水杨酸	57.57	20.644	±74.532
Betamethasone acetate	醋酸倍他米松	75.67	1.471	±1.094
Mometasone furoate	糠酸莫美他松	99.41	1.008	±0.594
Chlorothalonil	百菌清	40.95	1.971	±4.493
Perfluorobutanesulfonate (PFBS)	全氟丁基磺酸钾	96.74	0.842	±0.908
Perfluorobutanoic acid (PFBA)	全氟丁酸	79.23	0.227	±0.254
Perfluorodecanoic acid (PFDA)	全氟癸酸	64.39	1.546	±1.830
Perfluorododecanoic acid (PFDoDA)	全氟十二酸	62.02	0.743	±0.564
Perfluoroheptanoic acid (PFHpA)	全氟庚酸	67.06	0.120	±0.125
Perfluorohexanesulfonate (PFHxS)	全氟己基磺酸钠	100	2.975	±2.300
Perfluorononanoic acid (PFNA)	全氟壬酸	99.70	2.103	±1.565
perfluoro-*n*-pentanoic acid-1 (PFPeA)	全氟戊酸	98.81	3.996	±2.268
Perfluorooctanesulfonate (PFOS)	全氟辛烷磺酸	90.80	10.922	±7.973
Perfluorooctanoic acid (PFOA)	全氟辛酸	100	19.963	±11.887
Perfluoroundecanoic acid (PFUnDA)	全氟十一酸	99.41	1.394	±0.856
Sodium 4-chlorophenoxyacetate (4-CPA)	4-氯苯氧乙酸钠	78.93	5.594	±23.45
1*H*,1*H*,2*H*,2*H*-Perfluorooctanesulfonic acid	1*H*,1*H*,2*H*,2*H*-全氟辛烷磺酸	35.31	0.839	±0.293
Bis[2-(perfluorohexyl)ethyl] phosphate	双[2-(全氟己基)乙基]磷酸	48.66	1.066	±1.064
9-Chloro-3-oxa-perfluorononanesulfonic acid	9-氯-3-氧杂全氟壬烷磺酸钾	90.80	3.361	±3.098
Octisalate	水杨酸-2-乙基己基酯	76.26	0.477	±0.483
4-Hydroxybenzophenone	4-羟基二苯甲酮	52.52	0.078	±0.069
Monocyclohexyl phthalate	邻苯二甲酸单环己酯	87.54	0.417	±0.478
Monoethyl phthalate	邻苯二甲酸单乙酯	47.77	1.828	±2.101
DBPOB	3,5-二叔丁基-4-羟基苯甲酸	39.47	3.825	±3.363
Pyridate hydroxy type	哒草特	59.05	0.129	±0.067
Flumequine	氟甲喹	48.66	4.714	±3.378
Fipronil sulphone	氟虫腈砜	99.70	0.755	±0.508
Acesulfame potassium	安赛蜜	71.81	10.310	±30.662
Indole-3-butyric acid	吲哚乙酸	69.73	3.182	±2.513
Sucralose	三氯蔗糖	43.92	1.975	±1.974
Sodium saccharin	糖精钠	49.85	3.505	±15.701
Cyclamic acid	环拉酸	67.95	9.750	±22.408
3,5,6-Trichloro-2-pyridinol	3,5,6-三氯吡啶-2-醇	32.94	0.655	±0.783
Malathion dicarboxylic acid	马拉硫磷二羧酸	74.48	6.043	±1.414
2-Hydroxy-4-methoxybenzophenone	2-羟基-4-二苯甲酮-5-磺酸	52.52	7.024	±9.605
Diclofenac acid	双氯芬酸	37.98	2.856	±1.842

DBPOB: 3,5-di-*tert*-butyl-4-hydroxybenzoic acid.

### 2.3 高频检出化学物质残留的分布特征

55个高频检出物质包括全氟化合物、农兽药、塑化剂、食品添加剂、药物、紫外线吸收剂以及防腐剂和其他环境污染物等多个类别。人血清中高频检出外源性化学物质的平均水平为0.02~36.36 ng/mL,水杨酸、噻虫嗪、全氟辛酸、可的松和皮质醇5个物质的平均含量在15 ng/mL以上,分别为20.64、15.76、19.96、17.51以及36.36 ng/mL(图1a)。其中皮质醇不仅平均含量最高且检出率高达99.11%。本研究中持久性有机污染物全氟化合物的检测频率都在60%以上,且全氟辛酸的检出率高达100%,锦州地区样本的平均含量为19.96 ng/mL。全氟辛酸在人体中的暴露水平受到生活方式、居住环境及工作场所等多种因素的影响,例如沈阳人群为6.08 ng/mL^[19]^、上海人群为16.99 ng/mL^[20]^。

**图1:**
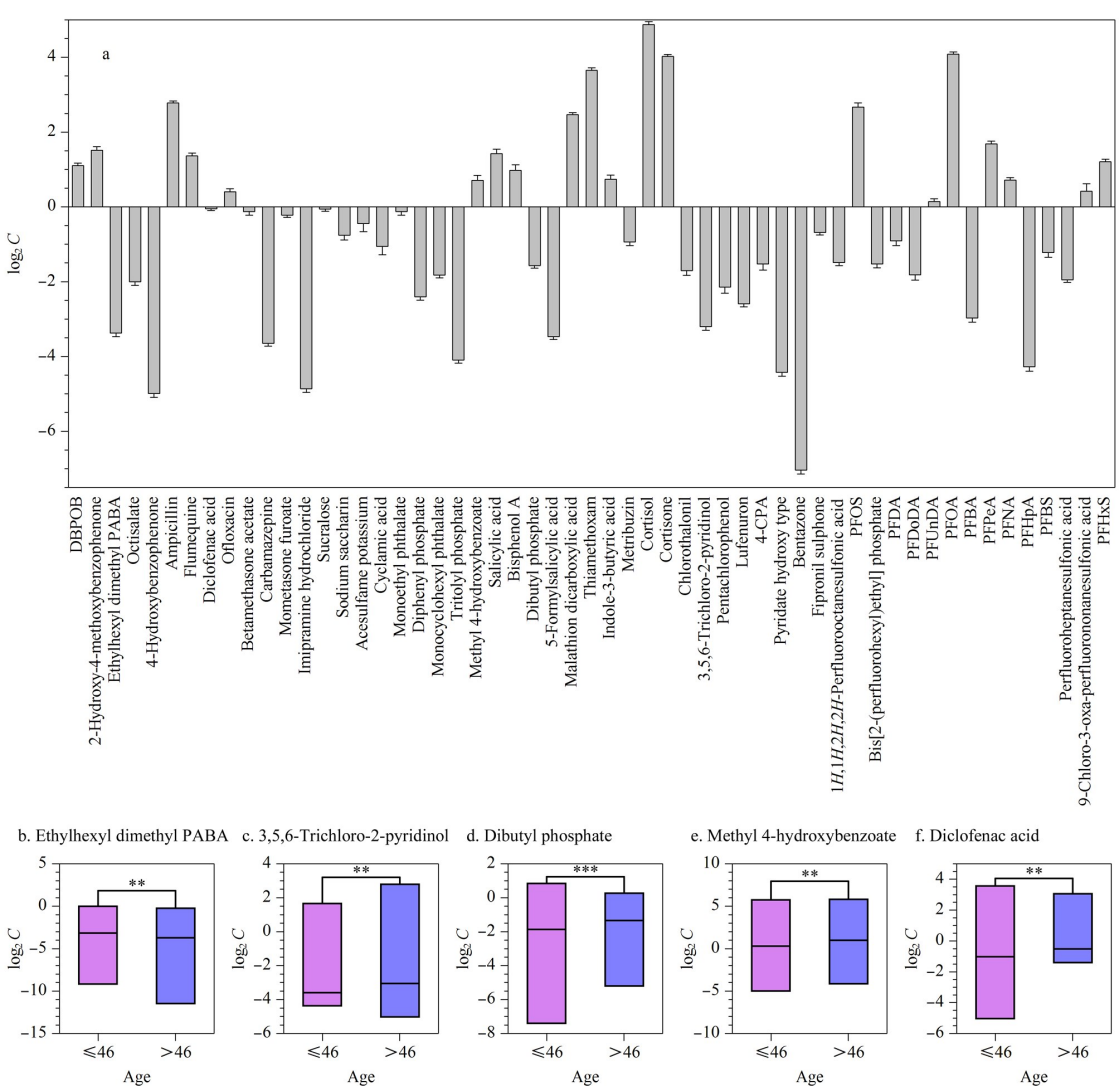
(a)高频检出暴露物质的浓度分布特征(*n*=337)以及(b~f)高频检出外源性化学物质的年龄分层

进一步探究了暴露物的年龄分布特征。根据全人群年龄中位数分为两组:≤46岁和>46岁。单变量分析发现,5种高频检出物质(二甲基PABA乙基己酯、3,5,6三氯吡啶-2-醇、磷酸二丁酯、尼泊金甲酯、双氯芬酸)具有年龄分布差异,其中二甲基PABA乙基己酯在≤46岁一组中含量较高(图1b), 3,5,6三氯吡啶-2-醇(图1c)、磷酸二丁酯(图1d)、尼泊金甲酯(图1e)、双氯芬酸(图1f)在>46岁一组中含量高。随着年龄的增加,外源性化学物质在体内蓄积增多,韩国的一项普通人群研究中也发现全氟烷基物质与年龄呈正相关^[21]^。

### 2.4 血清化学物质残留与MAFLD的风险分析

为探讨高频检出物与代谢相关脂肪性肝病发病风险的关联,采用二元逻辑回归评估污染物浓度的风险比(OR)。结果表明,马拉硫磷二羧酸和全氟壬酸可以增强单纯性脂肪肝患者的患病风险;全氟壬酸、全氟辛酸和全氟十一酸与合并一项代谢性疾病的脂肪肝患者的患病风险增加有关;氟虫腈砜、2-羟基-4-二苯甲酮-5-磺酸、双氯芬酸和尼泊金甲酯对合并两项代谢性疾病的脂肪肝患者起正相关作用;马拉硫磷二羧酸、尼泊金甲酯、安赛蜜以及4-羟基二苯甲酮能够增强合并三项代谢性疾病的脂肪肝患者的患病风险。这些风险物质在人群中的分布情况见图2,可以看出,与健康对照组相比,单纯性脂肪肝和合并代谢性疾病的脂肪肝患者体内的外源性化学物质含量不同程度的升高。越来越多的研究发现,长期暴露于环境污染物会增加代谢紊乱的风险^[22,23]^,与代谢相关脂肪性肝病发病率之间也存在正相关关系^[24]^。

**图2:**
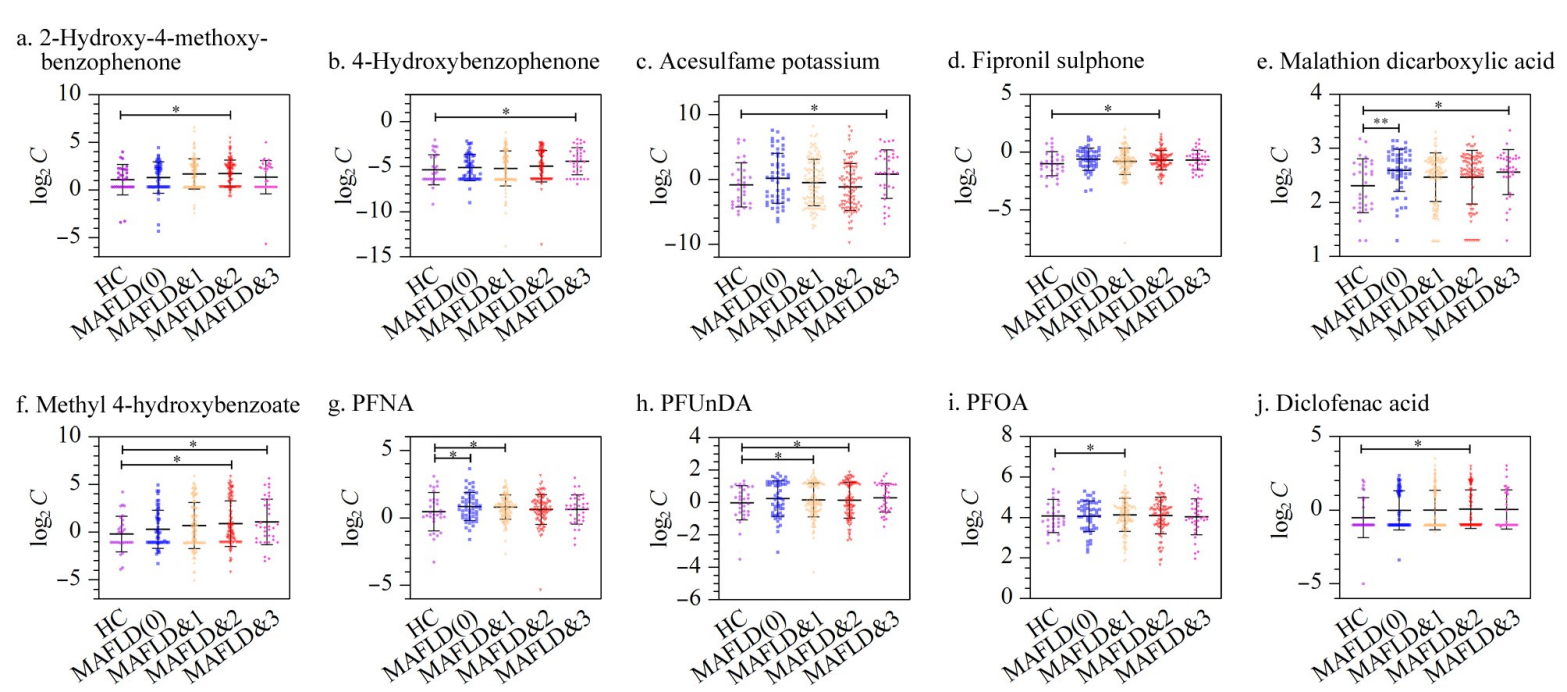
外源性化学风险物质残留的浓度分布图

校正性别和年龄混杂因素后,邻苯二甲酸单环己酯、氟虫腈砜和马拉硫磷二羧酸与单纯性脂肪肝患者的患病风险呈正相关;全氟辛酸、氟虫腈砜、全氟十一酸以及全氟壬酸可增强合并一项代谢性疾病脂肪肝患者的患病风险;只有氟虫腈砜与合并两项代谢性疾病的脂肪肝患者风险增加有关;3,5-二叔丁基-4-羟基苯甲酸、安赛蜜和4-羟基二苯甲酮可以增强合并三项代谢性疾病脂肪肝患者的患病风险(见图3)。

**图3:**
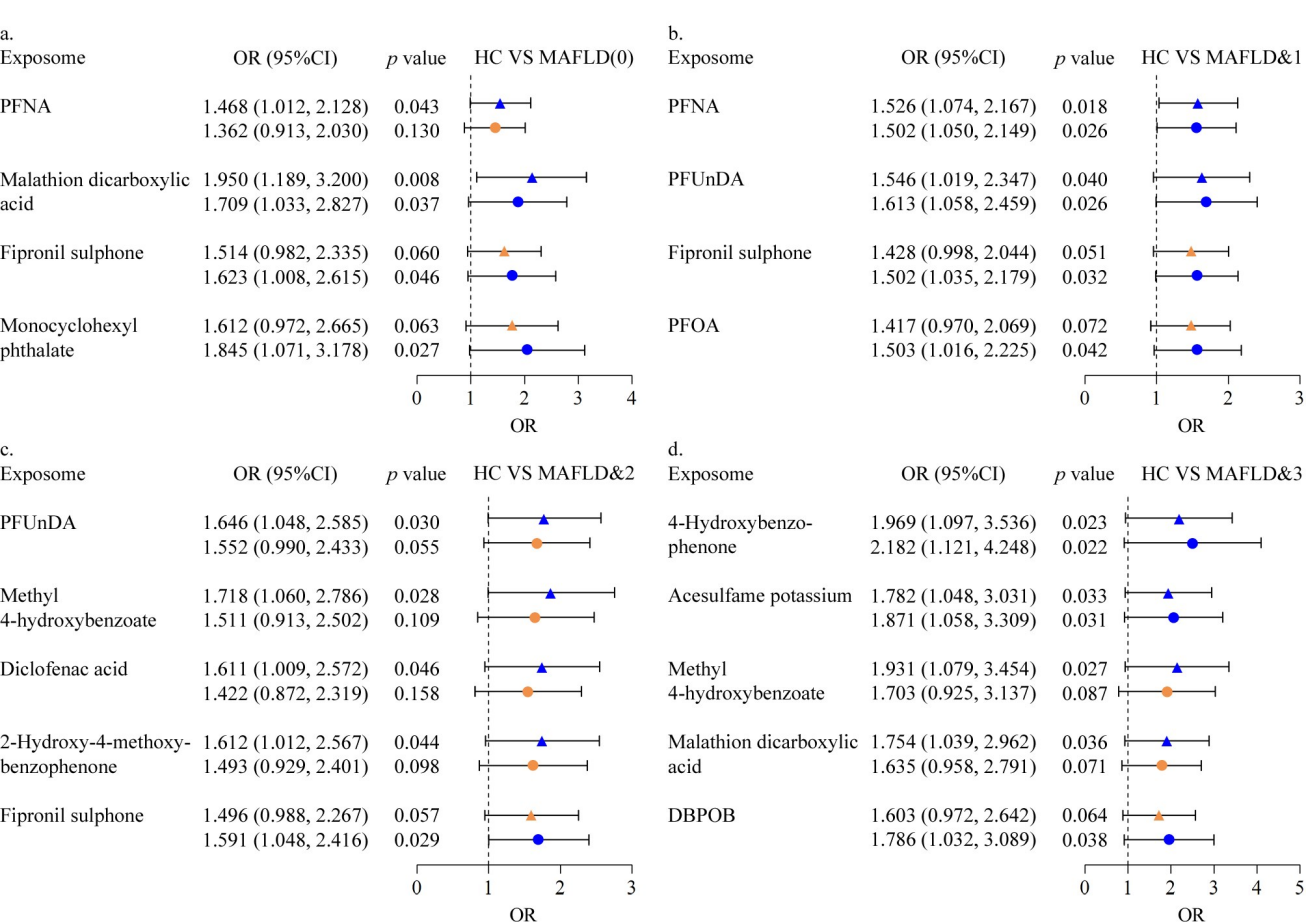
代谢相关脂肪性肝病风险OR值的森林图

流行病学研究报告显示,全氟化合物与高尿酸血症风险增加有关^[25,26]^, PFOA和PFNA与MAFLD的发展也有关系,全氟辛烷磺酸在结构上类似于脂肪酸,且能激活过氧化物酶体增殖物激活受体(PPARs)信号通路,进而扰乱脂质平衡^[27]^。同时,PFASs通过与血清白蛋白结合并转运至调节血清脂质水平的靶器官来影响血脂水平^[28]^。除了和MAFLD有关,PFASs暴露与肝纤维化的关系比脂肪变性更密切^[29]^。以往研究发现甜味剂果糖暴露可以加剧MAFLD的风险^[30]^,在本研究中,除全氟化合物外,还发现了农药马拉硫磷二羧酸、塑化剂邻苯二甲酸单环己酯以及食品添加甜味剂安赛蜜对MAFLD的风险呈正相关。邻苯二甲酸酯类暴露可能通过影响脂质信号通路,改变关键因子*FADS6*、*MVD*、*SC5D*、*PLA2G4E*的表达水平,导致肝脏细胞中出现脂肪蓄积^[31]^;根据现有研究推测,安赛蜜可能通过增加脱硫弧菌属的含量诱导炎症细胞因子的表达进而导致脂肪肝^[32]^。

### 2.5 基于UHPLC-HRMS的血脂谱分析

为了探究MAFLD脂质谱的扰动情况,利用脂质组学技术分析了MAFLD和健康对照的血清脂质组,共鉴定出879个脂质分子,主要有磷脂酰胆碱(PC)、磷脂酰乙醇胺(PE)、磷脂酰肌醇(PI)、溶血磷脂酰胆碱(LPC)、溶血磷脂酰乙醇胺(LPE)、脂肪酸(FA)、神经酰胺(Cer)、鞘磷脂(SM)、甘油三酯(TG)及甘油二酯等。峰面积经内标校正后,计算质控样本中的RSD,评价仪器的稳定性和数据的重复性。结果显示,超过92%的脂质其含量的RSD<30%,反映了仪器的稳定性和数据的可重复性。

有研究表明脂肪肝患者体内发生了脂代谢改变^[33]^,我们通过多变量分析进一步研究总体水平上的脂质紊乱,结果表明单纯性脂肪肝患者及脂肪肝同时伴有高尿酸血症、高血脂等代谢性疾病的患者体内均发生了脂质代谢异常。

### 2.6 代谢相关脂肪性肝病的脂质变化特征

通过单变量分析筛选代谢相关脂肪性肝病中发生扰动的脂质分子,在MAFLD(0)和其余组别中分别有383和330个差异脂质,有309个脂质分子在两组中均具有显著性。这些差异代谢物主要涉及Cer、TG、DG、PC、PE等亚类。由图4a可以明显看出,神经酰胺、甘油三酯和甘油二酯在代谢相关脂肪性肝病患者组中显著升高。本研究中代谢相关脂肪性肝病患者脂质紊乱情况与以往研究报道的结果一致^[34]^。

**图4:**
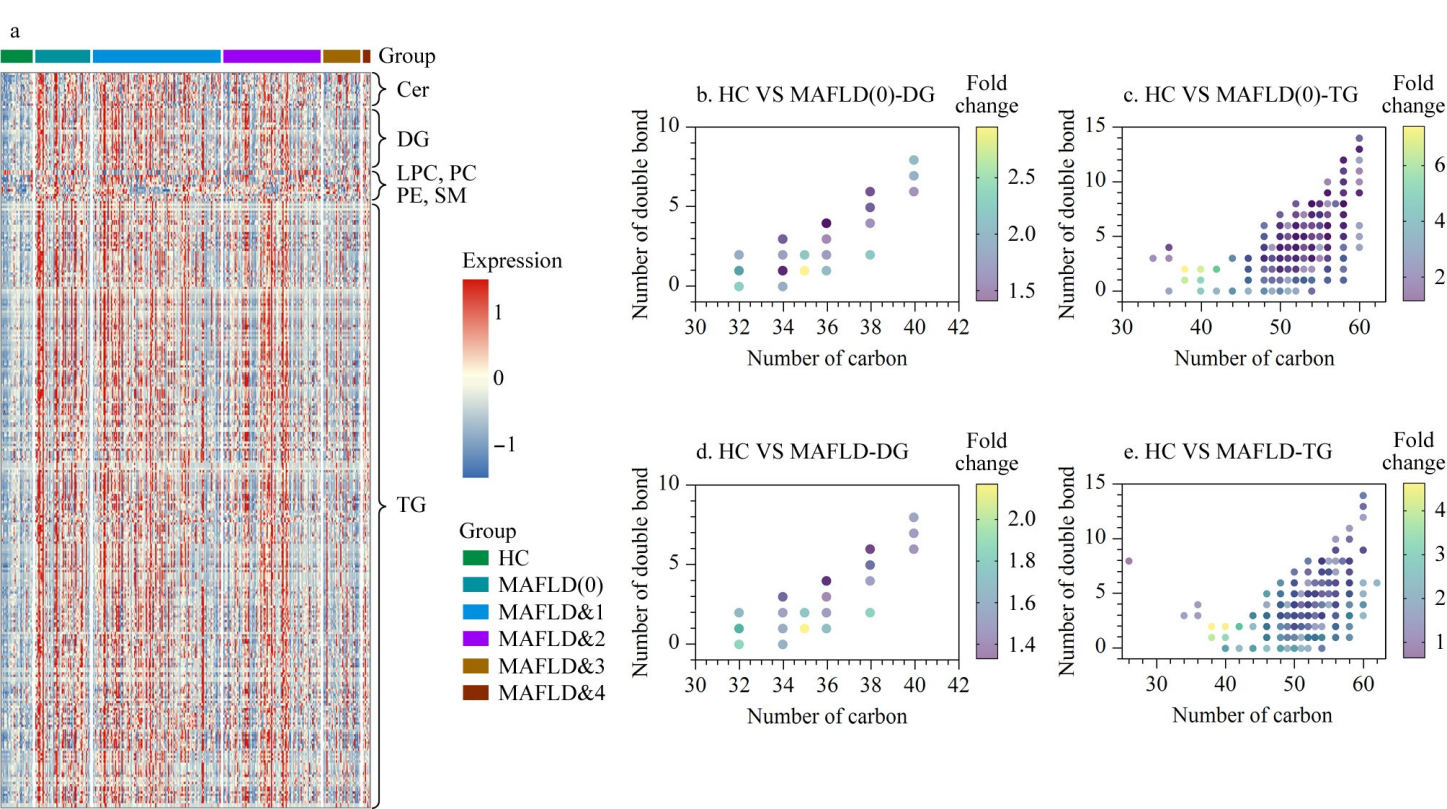
(a)差异脂质代谢物热图和(b~e)差异脂类的脂酰链和不饱和度信息

为了解脂质结构与疾病的关系,比较了差异脂质在脂酰链和不饱和度上的特点。结果表明,甘油二酯和甘油三酯在单纯性脂肪肝患者和合并代谢性疾病的脂肪肝患者组中的含量均高于健康对照。其中甘油二酯的酰基碳数主要集中在32~40,甘油三酯的酰基碳数主要集中在35~60,且均表现为多不饱和脂质分子的变化为主(图4b~e)。既往研究也发现碳数为50~54、双键数为7以下(大多为2、3)的TG与MAFLD^[35]^和2型糖尿病相关^[36]^,同时2型糖尿病的发病风险大小与MAFLD的严重程度平行,胰岛素抵抗、氧化应激、炎症反应和肠道菌群等是它们的共同病理机制,两者相互联系、互为因果^[37]^。

### 2.7 外源性化学物质残留引起与MAFLD风险相关的脂代谢扰动

为了探讨与暴露物相关的脂质代谢扰动,根据Spearman相关性分析来确定与MAFLD(0)风险相关的脂质代谢标志物。如图5a所示,分别有3、8和15个MAFLD(0)相关脂质代谢物与氟虫腈砜、邻苯二甲酸单环己酯以及马拉硫磷二羧酸的水平显著相关,其中TG与暴露物水平的相关性最强。SM(d18∶1-23∶0)和PE(18∶1p-22∶5)与氟虫腈砜呈显著正相关,并与MAFLD(0)风险增加有关。邻苯二甲酸单环己酯和马拉硫磷二羧酸主要与TG显著性最强,并与MAFLD(0)风险增加有关。

**图5:**
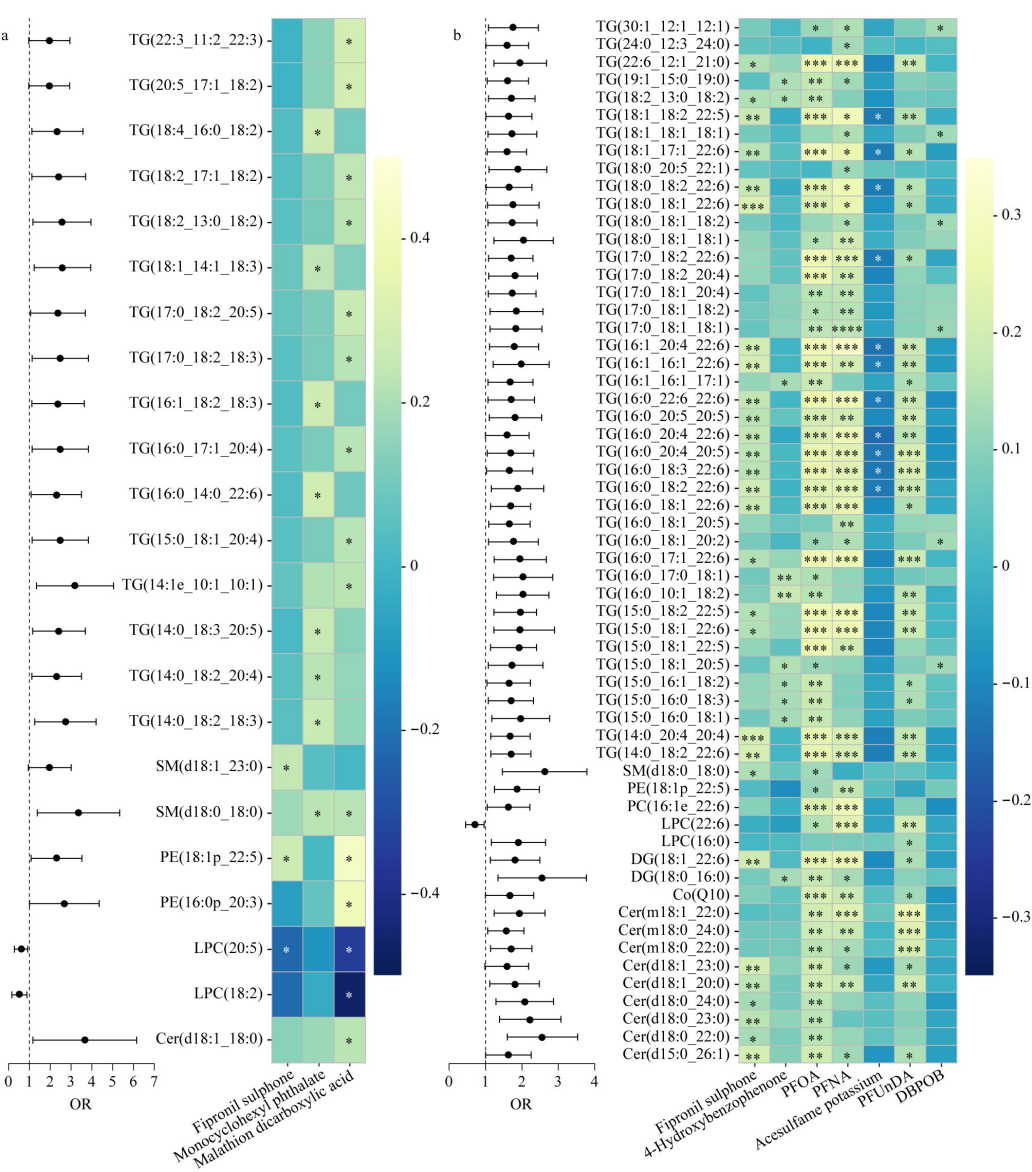
外源性化学物质残留引起的相关代谢物与(a)单纯性脂肪肝和(b)合并代谢性疾病脂肪肝风险的关系

有研究发现,农药杀虫剂对女性生殖健康有影响^[38]^,本研究发现了氟虫腈砜和马拉硫磷二羧酸对MAFLD的风险作用,氟虫腈砜因其较强的危害性,已被禁止使用。有动物实验发现,邻苯二甲酸二乙基己酯和邻苯二甲酸二丁酯具有显著诱发斑马鱼幼鱼脂肪肝风险的作用,邻苯二甲酸丁酯苯甲酯显著促进斑马鱼幼鱼的脂质沉积^[39]^。尽管基于人群MAFLD的邻苯二甲酸单环己酯的研究结果尚未有报道,本研究发现邻苯二甲酸酯类的暴露物邻苯二甲酸单环己酯对于MAFLD有风险作用。

在MAFLD的研究中,分别有110、31、192、137、18、34和8个MAFLD相关脂质代谢物分别与氟虫腈砜、4-羟基二苯甲酮、全氟辛酸、全氟壬酸、安赛蜜、全氟十一酸和3,5-二叔丁基-4-羟基苯甲酸的水平显著相关,其中甘油三酯与暴露物水平的相关性最强。大部分甘油三酯与氟虫腈砜、4-羟基二苯甲酮、全氟十一酸、全氟辛酸、全氟壬酸及3,5-二叔丁基-4-羟基苯甲酸水平呈显著正相关,并与MAFLD风险增加有关,与安赛蜜水平呈负相关,但与MAFLD风险增加有关。图5b展示了与每个外源性化学物质相关脂质中前10个最相关的(TOP10)脂质分子的MAFLD风险。

有研究发现,安赛蜜、阿斯巴甜、三氯蔗糖和糖精钠按最大使用量三元和四元联合使用所带来的健康风险是单独使用的2倍^[40]^。本研究发现,全氟化合物暴露与MAFLD的风险增加相关,既往研究^[41]^发现全氟化合物是过氧化物酶体增殖激活受体的激活剂,进而参与调节脂质代谢(如脂肪形成和储存等)。

以上结果表明,环境暴露物对于疾病的发生发展起到不容忽视的作用,从环境暴露角度深入了解MAFLD的发病机制值得关注。

## 3 结论

本研究表征了MAFLD患者血清中外源性化学物质的暴露特征和内源性脂质代谢物的扰动,并全面探讨了血清外源性化学暴露物与MAFLD的关系。结果表明,氟虫腈砜、马拉硫磷二羧酸和邻苯二甲酸单环己酯与单纯性脂肪肝的风险相关,氟虫腈砜、安赛蜜、全氟辛酸、全氟壬酸、全氟十一酸、4-羟基二苯甲酮以及3,5-二叔丁基-4-羟基苯甲酸与合并代谢性疾病脂肪肝的风险相关。MAFLD患者的神经酰胺、甘油三酯和甘油二酯较健康对照显著升高,其中TG与暴露和疾病的相关性最强,通过中间相遇原则发现,大多数效应标志物与外源性化学暴露物呈正相关,并且与人群的患病风险增加有关。我们的研究在脂质代谢水平上探讨了环境化学暴露对于代谢相关脂肪性肝病的影响,为后续更深入的机制研究提供了重要的科学依据。
